# Specific effects of bortezomib against experimental malignant pleural effusion: a preclinical study

**DOI:** 10.1186/1476-4598-9-56

**Published:** 2010-03-10

**Authors:** Ioannis Psallidas, Sophia P Karabela, Charalampos Moschos, Taylor P Sherrill, Androniki Kollintza, Sophia Magkouta, Panagiota Theodoropoulou, Charis Roussos, Timothy S Blackwell, Ioannis Kalomenidis, Georgios T Stathopoulos

**Affiliations:** 1 Applied Biomedical Research & Training Center "Marianthi Simou", Department of Critical Care & Pulmonary Services, General Hospital "Evangelismos", School of Medicine, National and Kapodistrian University of Athens, 3 Ploutarhou Str., 10675 Athens, Greece; 2 2nd Pulmonary Department, "Attikon" University Hospital, School of Medicine, National and Kapodistrian University of Athens, 1 Rimini Str., 12462 Haidari, Greece; 3 Division of Allergy, Pulmonary, & Critical Care Medicine, Vanderbilt University School of Medicine, Vanderbilt University Medical Center, T-1217 MCN, Nashville, TN 37232-2650, USA; 4 Department of Physiology, School of Medicine, University of Patras, Basic Medical Sciences Building, University Campus, 26504 Rio, Greece

## Abstract

**Background:**

We have previously shown that nuclear factor (NF)-*κ*B activation of mouse Lewis lung carcinoma (LLC) specifically promotes the induction of malignant pleural effusions (MPE) by these cells. In the present studies we hypothesized that treatment of immunocompetent mice with bortezomib tailored to inhibit cancer cell NF-*κ*B activation and not proliferation specifically inhibits MPE formation by LLC cells.

**Results:**

Treatment of LLC cells with low concentrations of bortezomib (100 ng/ml) inhibited NF-*κ*B activation and NF-*κ*B-dependent transcription, but not cellular proliferation. Bortezomib treatment of immunocompetent C57BL/6 mice bearing LLC-induced subcutaneous tumors and MPEs significantly blocked tumor-specific NF-*κ*B activation. However, bortezomib treatment did not impair subcutaneous LLC tumor growth, but was effective in limiting LLC-induced MPE. This specific effect was evidenced by significant reductions in effusion accumulation and the associated mortality and was observed with both preventive (beginning before MPE formation) and therapeutic (beginning after MPE establishment) bortezomib treatment. The favorable impact of bortezomib on MPE was associated with suppression of cardinal MPE-associated phenomena, such as inflammation, vascular hyperpermeability, and angiogenesis. In this regard, therapeutic bortezomib treatment had identical favorable results on MPE compared with preventive treatment, indicating that the drug specifically counteracts effusion formation.

**Conclusions:**

These studies indicate that proteasome inhibition tailored to block NF-*κ*B activation of lung adenocarcinoma specifically targets the effusion-inducing phenotype of this tumor. Although the drug has limited activity against advanced solid lung cancer, it may prove beneficial for patients with MPE.

## Background

A malignant pleural effusion (MPE) affects one in seven patients with cancer, most commonly lung or breast adenocarcinoma [1,2]. For these patients, MPE has severe consequences: it prohibits surgical cure and portends short and cumbersome survival [3]. Current treatments against MPE (pleural evacuation or pleurodesis) [1,2,4,5] are associated with hospital stay, interventional procedures, morbidity and mortality, and benefit only select patients [1-8]. Towards improving current practice against MPE, a better understanding of its pathogenesis is required, which may aid in developing effective strategies to block pleural fluid accumulation in patients with cancer.

The pathogenesis of MPE was unclear, mainly due to the lack of relevant animal models [9-11]. Using a prototype immune-competent mouse model, we described that activation of nuclear factor (NF)-*κ*B in lung adenocarcinoma specifically facilitates MPE, but not the growth or metastasis of this neoplasm [12,13]. We found that this effect of NF-*κ*B on MPE formation was not mediated via enhanced tumor growth, but by enhanced expression of NF-*κ*B-dependent gene products, including tumor necrosis factor (TNF) and C-C motif chemokine ligand (CCL) 2 [14,15]. We furthermore determined that this MPE-inducing phenotype of lung adenocarcinoma is not ubiquitous to all tumor types and involves specific MPE-associated phenomena such as inflammation, angiogenesis, and leakiness of pleural blood vessels [12-15]. These studies identified NF-*κ*B as a promising therapeutic target in lung adenocarcinoma-induced MPE.

Various approaches have been tailored to block NF-*κ*B in cancer cells, since the transcription factor has emerged as a promoter of inflammation-associated cancers [16-18]. These include blockade of inhibitor of NF-*κ*B (I*κ*B) kinases (IKK) [19] and proteasome inhibition, which suppresses NF-*κ*B by diminishing I*κ*B degradation [20]. Although the latter approach is less specific, the proteasome inhibitor bortezomib blocks NF-*κ*B in a variety of cells and is already in clinical use against multiple myeloma [20-22]. Unfortunately, the initial optimism regarding activity of the drug against non small-cell lung cancer (NSCLC) [23] that led to several completed/ongoing phase I/II trials was recently hampered by the results of these trials reporting limited or no single-agent activity of bortezomib against advanced NSCLC [24,25].

In the present studies we hypothesized that bortezomib treatment tailored to inhibit NF-*κ*B activation of Lewis lung cancer (LLC) is specifically effective in limiting MPE, but not solid tumor formation by this neoplasm. We tested our hypothesis by titrating the effects of bortezomib on LLC cell NF-*κ*B activation and proliferation and by conducting parallel experiments of intrapleural and subcutaneous introduction of this tumor into syngeneic mice.

## Methods

### Reagents

Bortezomib (Millenium, Cambridge, MA) was purchased from the pharmacy, D-luciferin from Biosynth AG (Naperville, IL), 3-(4,5-dimethylthiazol-2-yl)-5-(3-carboxymethoxyphenyl)-2-(4-sulfophenyl)-2H-tetrazolium, inner salt (MTS) assay, passive lysis buffer, and firefly luciferase assay system from Promega (Madison, WI), recombinant human (rh) TNF from Peprotech (London, UK), ELISA from Peprotech (London, UK) and R&D Systems (Minneapolis, MN), anti-proliferating cell nuclear antigen (PCNA) antibody from SantaCruz Biotechnology (Santa Cruz, CA), terminal deoxynucleotidyl nick-end labeling (TUNEL) kit from Roche (Penzberg, Germany), anti-factor VIII-associated protein (F8A) antibody from Invitrogen (San Francisco, CA), and Evans' blue from Sigma (St Louis, MO).

### Cell experiments

LLC mouse lung adenocarcinoma cells were purchased from the American Type Culture Collection (Manassas, VA; identifier: CRL-1642), were verified by antigen H-2b expression, and were stored at -80°C, resuscitated, and cultured at 37°C in 5% CO2-95% air using DMEM 10% FCS supplemented with glutamine and 100 mg/l penicillin/streptomycin. Wild-type or *pNGL *LLC cells stably expressing a NF-*κ*B reporter (NF-*κ*B. GFP.LUC; *pNGL*) [12-14] were plated at equal densities in 6- or 96-well culture dishes till 20-30% confluent and were incubated with PBS or 1 nM rhTNF, in the presence of varying concentrations of bortezomib. Luciferase activity was determined after four hours by luciferase assay and bioluminescent imaging, as described below. Cell viability and mediator elaboration into culture media were determined after 24 hours, using MTS reduction capacity and ELISA, respectively. All cell experiments were done in triplicate.

### Determination of luciferase bioactivity

Luciferase activity of whole cells or tissue homogenized in passive lysis buffer (Promega, Madison, WI) was determined using a "Junior" luminometer (EG&G Berthold, Bad Wildbad, Germany), after addition of 100 μl firefly luciferase assay system to 20 μl sample, as described previously [14]. Luciferase activity was corrected for total protein, determined using the Bio-Rad assay (Hercules, CA). Serial bioluminescence imaging of live mice bearing *pNGL *LLC-induced subcutaneous tumors or MPE was done using intravenous injection of 1 mg D-luciferin, and of cultured *pNGL *LLC cells four hours after treatment application using addition of 10 mM D-luciferin [12,13]. Imaging was performed using the Xenogen IVIS cooled CCD (Xenogen, Alameda, CA). Data were analyzed using Living Image v.2.50 (Xenogen) and IgorPro (Wavemetrics, Lake Oswego, OR) [12,13].

### Cytokine determinations

Mouse TNF, C-X-C motif chemokine ligand (CXCL) 1, CXCL2, and CCL2 (detection limits: 5.1, 7.8, 1.5, and 15.6 pg/ml, respectively) expression levels were determined using ELISA and were corrected for total protein.

### Animal Models

C57BL/6 mice were purchased from the Hellenic Pasteur Institute (Athens, Greece) and the Jackson Laboratory (Bar Harbor, ME) and inbred at the Animal Care facilities of the General Hospital Evangelismos (Athens, Greece) and of Vanderbilt University. Animal care and experiments were approved by the Veterinary Administration, Prefecture of Athens, Greece, and the Institutional Animal Care and Use Committee at Vanderbilt University and were conducted according to international standards http://grants.nih.gov/grants/olaw/GuideBook.pdf. Experimental mice were sex-, weight (19-24 g)-, and age (8-10 week)-matched. Solid adenocarcinomas and MPEs were generated, respectively, by injections of wild-type or *pNGL *LLC cells in the flank (5 × 10^5 ^cells) or pleural space (1.5 × 10^5 ^cells). In mice with subcutaneous LLC tumors, three vertical tumor dimensions (δ1, δ2, δ3) were measured weekly, and tumor volume (V) was determined using the formula V = π × (δ1 × δ2 × δ3)/6. In mice with LLC-induced MPEs, sacrifice, necropsy, and specimen (MPE, blood, tumor) collection were performed on day 14 as described previously [12-15,26].

### *In vivo *bortezomib treatment

Mice with subcutaneous LLC tumors received bi-weekly intraperitoneal bortezomib (100 ng/g = 0.1 mg/kg in 100 μl PBS) or 100 μl PBS control at days 2, 5, 9, 12, 16, 19, 23, and 26 after tumor cell inoculation. Mice that received intrapleural LLC cells were treated with intraperitoneal bortezomib (100 ng/g = 0.1 mg/kg in 100 μl PBS) or 100 μl PBS control starting either before (bortezomib prevention trial; days 2, 5, 9, and 12 after LLC cells), or after the onset of active fluid exudation (bortezomib regression trial; days 9 and 12 after LLC cells), which occurs at day 8 in the LLC-induced mouse MPE model [12,26].

### Histology & cytology

Tumors were fixed in 10% neutral buffered formalin (24 hours) and 70% ethanol (3 days). Tumors were dissected and embedded in paraffin. 5-μm-thick sections were cut, mounted on glass slides, and stained with hematoxylin & eosin. Alternatively, tissue sections were immunostained using antibodies for PCNA, TUNEL, and F8A, and immune-labeling was quantified, as described previously [12-15,26]. For cytocentrifugal specimen (cytospin) preparation, 50000 pleural fluid cells were used. The slides were air dried, fixed in methanol for 10 seconds, and stained with May-Gruenwald-Giemsa. Distinct cell types were enumerated as a percentage of 400 cells on the slide.

### Pleural Permeability Assay

At day 14 post-LLC cells, mice bearing MPEs induced by LLC cells received 200 μl of 4 mg/ml Evans' blue solution (0.8 mg) intravenously and were killed 1 hour later. Pleural fluid Evans' blue concentration was determined using absorbance at 605 nm, as described previously [12-15,26].

### Statistics

All values are presented as mean ± standard error of mean (SEM). Survival is given as median (95% confidence interval) and was analyzed using Kaplan-Meier estimates. Differences in the means between two or multiple groups were examined using the Student's t-test or one-way ANOVA with least square difference post-hoc tests for normally distributed data, and the Mann-Whitney u-test or Kruskal-Walis test with Mann-Whitney u-post-hoc tests for not normally distributed data. Differences in survival were assessed by log-rank test. All *P *values are two-tailed; *P *values < 0.05 were considered significant. All statistical analyses were performed using the Statistical Package for the Social Sciences v.13.0.0 (SPSS, Chicago, IL).

## Results

### Tailoring proteasome inhibition to block NF-κB activation of lung adenocarcinoma *in vitro *and *in vivo*

**Figure 1:**
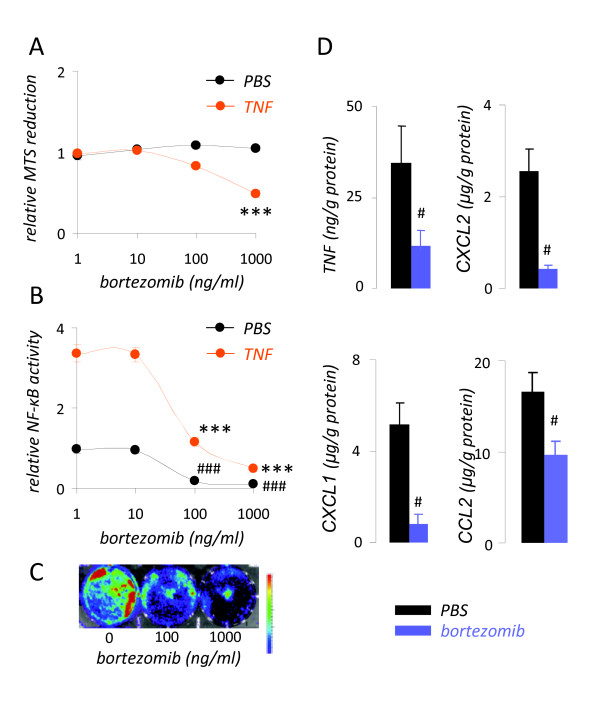
**Tailoring of bortezomib treatment to target nuclear factor (NF)-*κ*B activation of mouse lung adenocarcinoma cells *in vitro***. *(A) *Relative MTS reduction capacity of Lewis lung cancer (LLC) cells after 24 hours of incubation with or without 1 nM recombinant human (rh) TNF in the presence of varying concentrations of bortezomib. *(B) *Relative nuclear factor (NF)-*κ*B-dependent luciferase expression of NF-*κ*B. GFP.luc (*pNGL*) LLC cells after 4 hours of incubation with or without 1 nM rh TNF in the presence of varying concentrations of bortezomib. *(C) *Bioluminescence image of *pNGL *LLC cells exposed for 4 hours to the indicated doses of bortezomib. Colors on the top of the scale indicate high light emission. *(D) *Cytokine/chemokine release by wild-type LLC cells incubated with or without 100 ng/ml bortezomib for 24 hours. TNF, tumor necrosis factor; CXCL, C-X-C motif chemokine ligand; CCL, C-C motif chemokine ligand. All experiments were done thrice. *Columns, dots*, mean; *bars*, standard error of mean. # and ###: *P *< .05 and .001, respectively, compared with cells treated with PBS. * and ***: *P *< .05 and .001, respectively, compared with cells treated with 1 nM rhTNF.

Initially we sought to titrate the effects of bortezomib treatment on cellular proliferation and NF-*κ*B activation of LLC cells *in vitro*. LLC cell proliferation was not suppressed by bortezomib alone, but only when high concentrations of the proteasome inhibitor were combined with exposure to TNF (Figure 1A). Using a NF-*κ*B-reporter (*pNGL*), we determined that bortezomib inhibited constitutive and TNF-inducible NF-*κ*B activation of LLC cells at much lower concentrations (100 ng/ml) (Figures 1B,1C). At these low concentrations, bortezomib also inhibited NF-*κ*B-dependent gene transcription, as determined by TNF, CXCL1, CXCL2, and CCL2 elaboration (Figure 1D). These results indicated that low concentrations of bortezomib can be used to limit mouse lung adenocarcinoma NF-*κ*B activation without affecting cellular proliferation and identified 100 ng/ml as a suitable concentration for this purpose.

**Figure 2:**
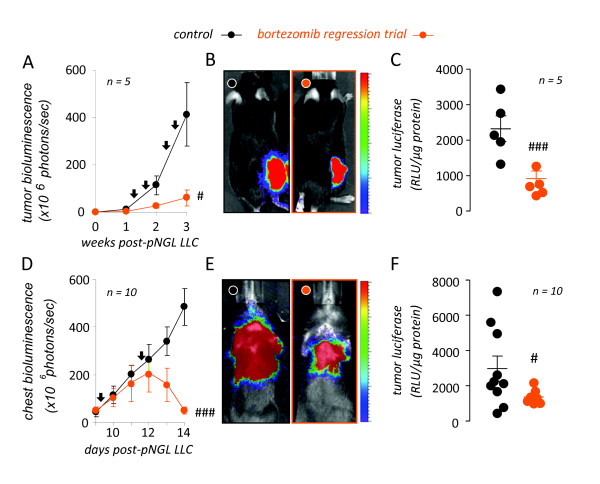
**Tailored bortezomib treatment inhibits tumor-specific nuclear factor (NF)-*κ*B activation of subcutaneous and intrapleural lung adenocarcinoma**. *(A-C) *NF-*κ*B-targeted bortezomib treatment blocks NF-*κ*B activation of subcutaneous adenocarcinoma *in vivo*. Wild-type C57BL/6 mice received 5 × 10^5 ^subcutaneous NF-*κ*B-reporter (NF-*κ*B. GFP.luc, *pNGL*) Lewis lung carcinoma (LLC) cells and were allowed one week for tumors to implant, where after they received bi-weekly intraperitoneal bortezomib (100 ng/g = 0.1 mg/kg) or PBS in a regression trial. Mice were serially imaged for bioluminescence after intravenous injection of 1 mg D-luciferin at the time-points indicated *(A, B) *and were sacrificed on day 21 for determination of NF-*κ*B-dependent luciferase bioactivity of tumor tissue homogenates *(C)*. Shown are time course of tumor-specific NF-*κ*B activity in flank tumors *(A)*, representative bioluminescence images at day 21 *(B)*, and summary of luciferase assay data obtained at day 21 *(C). (D-F) *NF-*κ*B-targeted bortezomib treatment blocks NF-*κ*B activation of intrapleural adenocarcinoma *in vivo*. C57BL/6 mice received 1.5 × 10^5 ^intrapleural *pNGL *LLC cells and were allowed one week, where after they received bi-weekly bortezomib (100 ng/g = 0.1 mg/kg) or PBS. Mice were imaged for bioluminescence *(D, E) *and were sacrificed on day 14 for determination of NF-*κ*B-dependent luciferase bioactivity of tumor tissue *(F)*. Shown are time course of tumor-specific NF-*κ*B activity in MPE-bearing mice *(D)*, representative bioluminescence images at day 14 *(E)*, and summary of luciferase assay data obtained at day 14 *(F)*. *n*, sample size; *RLU*, relative light units. *Dots*, mean (left) or raw data points (right); *lines in panels on the right*, mean; *bars*, standard error of mean. # and ###: P < .05 and .001, respectively, compared with control.

Based on the good bioavailability of bortezomib [27], we extrapolated the above studies to *in vivo *experiments of low-dose (100 ng/kg = 0.1 mg/kg) bortezomib treatment of C57BL/6 mice bearing LLC-induced subcutaneous tumors or MPEs, aiming to block tumor cell NF-*κ*B activation without affecting cell proliferation. To monitor the effects of proteasome inhibition specifically on tumor cell NF-*κ*B activation, we employed models of subcutaneous or intrapleural delivery of NF-*κ*B-luciferase reporter (*pNGL*) LLC cells to wild-type C57BL/6 mice [12-14]. Following reporter cell injection, subcutaneous or intrapleural tumors were allowed one week to form, and mice started receiving bi-weekly control or bortezomib treatment on the second week of the experiments. Tumor NF-*κ*B activation was monitored by serial bioluminescence imaging and by luciferase activity determinations in tumor tissues [12-14]. These studies showed that this bortezomib regimen was effective in suppressing tumor-specific NF-*κ*B activation of established subcutaneous and pleural tumors (Figure 2).

### Specific suppression of lung adenocarcinoma-induced MPE formation but not tumor growth by NF-κB-targeting bortezomib treatment

**Figure 3:**
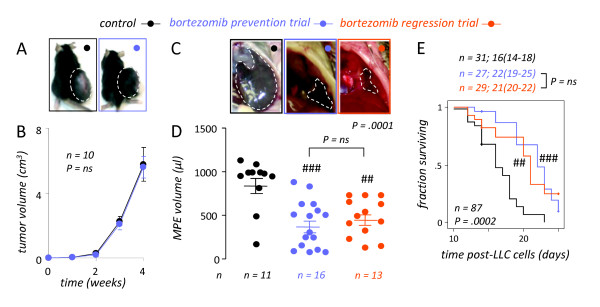
**NF-*κ*B-targeted bortezomib treatment specifically inhibits MPE but not subcutaneous tumor formation by lung adenocarcinoma**. *(A, B*) NF-*κ*B-targeted bortezomib treatment has no effect on subcutaneous Lewis lung carcinoma (LLC) growth. C57BL/6 mice received 5 × 10^5 ^subcutaneous LLC cells followed by bi-weekly intraperitoneal bortezomib (100 ng/g = 0.1 mg/kg) or PBS. Separate subsets of mice received treatment either immediately (prevention trial) or starting one week after tumor implant (regression trial). Tumor volume was determined weekly and mice were sacrificed on day 28. Representative images at 4 weeks (*A; *dashed lines outline tumors) and mean volume *(B) *of subcutaneous tumors in PBS and bortezomib treated mice. Results from prevention and regression trial were identical and were grouped for demonstration purposes. *Dots*, mean; *bars*, standard error of mean. *(C-E*) NF-*κ*B-targeted bortezomib treatment is effective against malignant pleural effusion (MPE) induced by LLC cells. C57BL/6 mice received 1.5 × 10^5 ^intrapleural LLC cells followed by bi-weekly bortezomib (100 ng/g = 0.1 mg/kg) or PBS. Mice were enrolled in prevention (immediate treatment) or regression (treatment starting after one week) trials and were sacrificed on day 14 *(C, D) *or observed till moribund *(E)*. *(C) *Representative transdiaphragmatic photographs at 14 days (dashed lines outline MPEs) and *(D) *mean volume of MPE in PBS- and bortezomib-treated mice. *Dots*, raw data points; *lines*, mean; *bars*, SEM. *(E) *Survival of MPE-bearing mice treated with PBS or bortezomib. Shown are Kaplan-Meier survival curves and estimates (median, 95% confidence interval) of pooled data from three independent experiments. *(D, E) *Note that results from prevention and regression trials were not significantly different (ns). *n*, sample size; *P*, probability values. ## and ###: P < .01 and .001, respectively, compared with control.

We subsequently sought to assess the impact of NF-*κ*B-tailored bortezomib treatment on LLC growth and MPE induction *in vivo*. For this, the above animal experiments were repeated using injection of wild-type LLC cells into the subcutaneous or pleural space of C57BL/6 mice. In accord with our hypothesis, treatment of C57BL/6 mice bearing subcutaneous LLC tumors with bi-weekly intraperitoneal bortezomib at 100 ng/g had no effect on tumor growth compared with PBS control treatment (Figures 3A,3B). In stark contrast, bortezomib-treated mice bearing LLC-induced MPEs showed significantly improved indices of MPE control, including MPE volume and survival, compared with PBS-treated mice with MPEs (Figures 3C-E). Importantly, preventive administration of the drug (*ie*, starting prior to MPE development) did not confer any additional protection against MPE formation compared with therapeutic treatment of MPE-bearing mice starting after MPE establishment. These results indicated that our low-dose proteasome inhibition regimen designed to block NF-*κ*B activation of LLC lung adenocarcinoma had no effect on the growth of this tumor in solid form, but was highly effective against already established LLC-induced MPE in mice.

**Figure 4:**
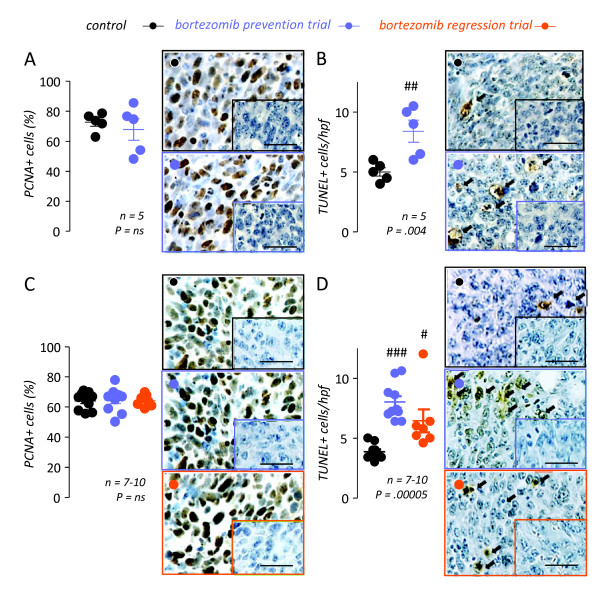
**NF-*κ*B-targeted bortezomib treatment does not impact tumor cell proliferation in vivo**. Subcutaneous *(A, B) *and pleural *(C, D) *tumor tissue was obtained on days 28 and 14, respectively, from experiments described in Figure 3 and was immunostained for proliferating cell nuclear antigen (PCNA; *A, C*) and terminal deoxynucleotidyl nick-end labeling (TUNEL; *B, D*), indicators of cell proliferation and apoptosis, respectively. Shown are summary of results and representative photomicrographs (Å = 600; scale bars = 50 μm). NF-κB-targeted bortezomib treatment had no effect on Lewis lung carcinoma (LLC) cell proliferation in subcutaneous or pleural tumors *(A, C)*. However, bortezomib treatment resulted in enhanced apoptosis rates in both tumor sites *(B, D)*. *n*, sample size; *P*, probability values; *hpf*, high-power field. *Dots*, raw data points; *lines*, mean; *bars*, standard error of mean. #, ##, and ###: P < .05, .01, and .001, respectively, compared with control.

To corroborate these findings of specific MPE, but not tumor growth suppression by NF-*κ*B-tailored bortezomib treatment, we next assessed the proliferation and apoptosis of tumor cells *in vivo *using PCNA and TUNEL labeling, respectively (Figure 4). In both subcutaneous and pleural LLC-induced tumors, bortezomib treatment had no effect on cell proliferation compared with PBS control treatment. On the contrary, tumor cell apoptosis rates were modestly but significantly increased in both subcutaneous and pleural tumors from bortezomib-treated mice, a finding consistent with the aforementioned NF-*κ*B inhibitory effects of the employed bortezomib regimen. These results confirmed that the specific anti-MPE effects of NF-*κ*B-tailored proteasome inhibition were not linked with tumor growth inhibition. Moreover, the results of TUNEL suggested that the anti-MPE effects of bortezomib-mediated NF-*κ*B blockade could neither be ascribed to induction of tumor cell apoptosis, since a pro-apoptotic response of equal magnitude was observed in MPE-associated and subcutaneous LLC tumors.

### NF-κB-tailored proteasome inhibition down-regulates proinflammatory gene expression of pleural tumors and cardinal MPE-associated phenomena

**Figure 5:**
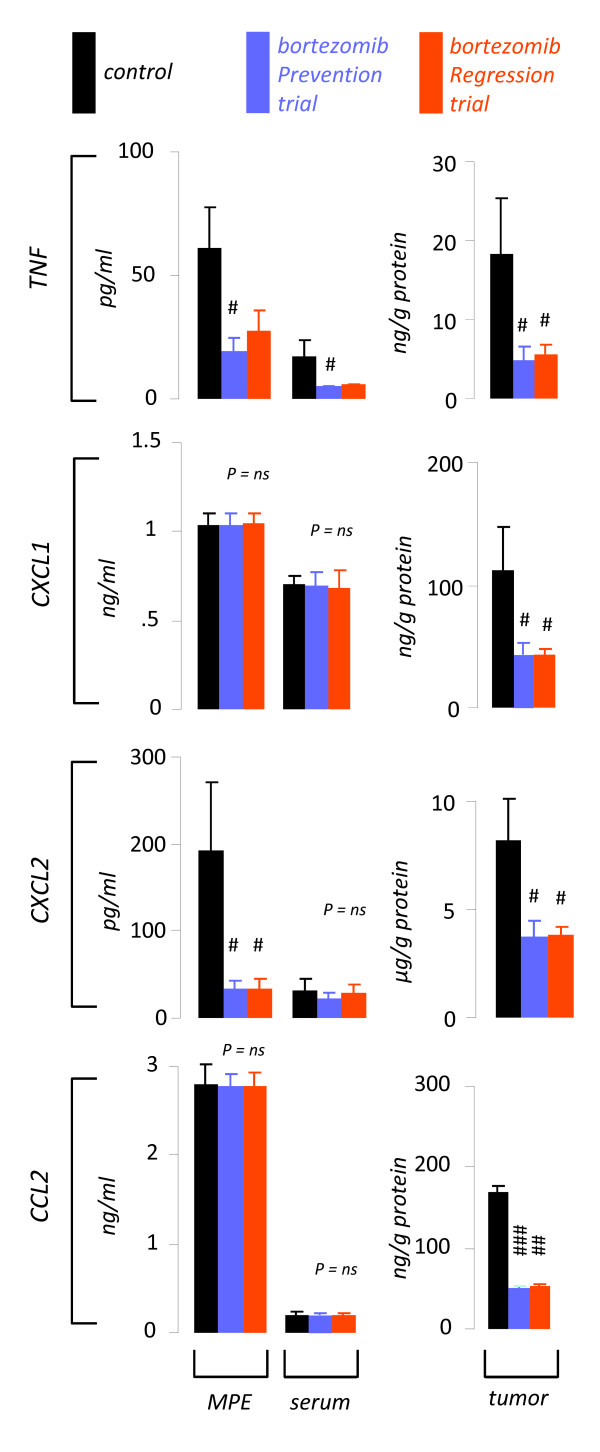
**Tailored bortezomib treatment down-regulates NF-*κ*B-dependent paracrine mediator expression of pleural lung adenocarcinoma**. Pleural fluid, blood, and pleural tumor tissue NF-*κ*B-dependent gene product levels were determined in samples obtained at day 14 from mice with MPE enrolled in control *(n = 10)*, bortezomib prevention *(n = 10)*, and bortezomib regression *(n = 7) *trials as in Figure 3C-D. TNF, tumor necrosis factor; CXCL, C-X-C motif chemokine ligand; CCL, C-C motif chemokine ligand. *Columns*, mean; *bars*, standard error of mean; *n*, sample size; *P*, overall probability values. #, ## and ###: P < .05, .01, and .001, respectively, compared with control.

Based on the above results, we postulated that the specific effects of NF-*κ*B-targeted bortezomib treatment against MPE are mediated via suppression of NF-*κ*B-dependent gene expression, a hypothesis supported by previous work of our group [12-15]. Indeed, TNF and CXCL2 were decreased in MPEs from bortezomib-treated mice. In addition, TNF was down-regulated in the blood from bortezomib-treated mice. However, all pro-inflammatory NF-*κ*B-dependent mediators assayed were consistently decreased in pleural tumor tissue from bortezomib-treated mice compared with specimens obtained from PBS-treated mice (Figure 5). The results obtained from tumor tissue mirrored bortezomib-induced suppression of the expression of these mediators by LLC cells *in vitro *(Figure 1B) and, in addition to the TUNEL results described above, served as biomarkers of efficient *in vivo *suppression of NF-*κ*B activation of mouse lung adenocarcinoma.

**Figure 6:**
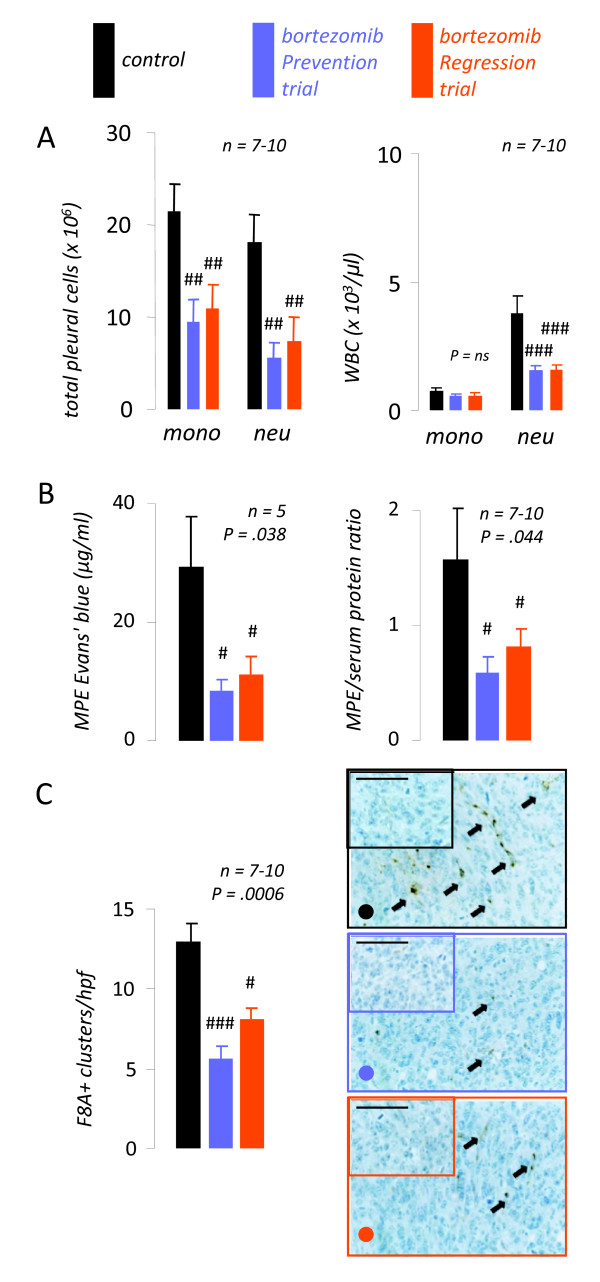
**NF-*κ*B-targeted bortezomib inhibits malignant pleural effusion (MPE)-associated inflammation, vascular leakiness, and angiogenesis**. Mice were treated as in Figure 3C-D. *(A) *Pleural fluid and blood inflammatory cell numbers at day 14. *(B) Left: *MPE Evans' blue levels determined at day 14, one hour after intravenous delivery of 0.8 mg of the dye. *Right: *MPE/serum protein ratio. Both Evans' blue leakage and protein ration are indicators of vascular hyperpermeability. *(C) *Pleural tumor tissue microvessel density assessed by immunoreactivity for factor VIII-associated protein (F8A). Summary of data *(left) *and representative microphotographs (*right*; Å = 400; scale bar = 100 μm; *arrows*, new vessels). MPE, malignant pleural effusion; *WBC*, white blood cells; *mono*, mononuclear cells; *neu*, neutrophils; *hpf*, high-power field. *Columns*, mean; *bars*, standard error of mean; *n*, sample size; *P*, overall probability values. #, ## and ###: P < .05, .01, and .001, respectively, compared with control.

Finally, we examined the effects of NF-*κ*B-targeted bortezomib treatment on inflammation, vascular hyperpermeability, and new vessel formation, mechanisms central in MPE formation that are profoundly influenced by the tumor cells' mediator expression profile [1,2,9-15,26]. Cytologic determinations revealed significantly reduced numbers of neutrophils in MPE and blood from bortezomib-treated mice, compared with controls (Figure 6A). In addition, mononuclear cell numbers in MPEs from bortezomib-treated mice were significantly lower compared with controls. To determine vascular hyperpermeability, separate sets of experimental mice received 0.8 mg intravenous albumin-binding Evans' blue one hour before sacrifice (day 14). Evans' blue leak from the bloodstream into MPE was significantly lower in bortezomib-treated mice compared with controls, indicating reduction of albumin leakage (Figure 6B, left). An additional marker of pleural vascular permeability, the pleural fluid/serum protein ratio [26], was also reduced by bortezomib treatment (Figure 6B, right). Finally, pleural tumor tissue immune-labeling for the endothelial marker F8A revealed significant reductions in microvessel density of pleural tumors from bortezomib-treated mice (Figure 6C). Collectively, the above findings suggested that NF-*κ*B-tailored proteasome inhibition in the mouse model of adenocarcinoma-induced MPE suppresses the gene expression of NF-*κ*B-dependent mediators in pleural LLC tumors, which are required for the paracrine promotion of inflammation, vascular leakiness, and angiogenesis, the major known pathways for MPE induction by cancer cells.

## Discussion

In the present studies we examined the effects of the proteasome inhibitor bortezomib on NF-*κ*B-dependent MPE induction by lung adenocarcinoma. By tailoring drug treatment to specifically target NF-*κ*B activation and not cell proliferation of this tumor we achieved consistent blockade of the transcription factor in cancer cells *in vitro *and *in vivo*. Delivered in this fashion, the proteasome inhibitor limited MPE formation by LLC cells without impacting their subcutaneous growth rate. In addition, therapeutic (*eg*, initiated before MPE formation) proteasome inhibition was equally effective in achieving MPE control and in prolonging survival with preventive treatment (*eg*, initiated after MPE establishment), suggesting specific anti-MPE effects of NF-*κ*B-targeted bortezomib treatment. Indeed, tailored bortezomib treatment suppressed NF-*κ*B-dependent gene expression of lung adenocarcinoma *in vitro *and *in vivo*, resulting in down-regulation of all major paracrine phenomena known to be involved in MPE formation: inflammation, vascular hyperpermeability, and neoangiogenesis.

This is the first preclinical study of the effects of any NF-*κ*B or proteasome inhibitor on MPE. Our results indicate that bortezomib is highly effective against experimental MPE, favorably impacting all outcome measures of MPE control. In addition, the drug was effective even when given after the onset of active lung adenocarcinoma-induced fluid exudation into the pleural space, implying the potential for therapeutic use against already established MPE. Human MPE is a significant clinical problem, a fact reflected by the overt insufficiency of current treatments [8] and by expert recommendations for upgrading its prognostic significance in the TNM staging system for NSCLC from a T4 to a M1a descriptor [28]. In face of the above, our findings may prove clinically useful against human MPE.

In addition to their potential clinical utility, our results yield insights into the determinants of site-specific lung adenocarcinoma progression. In this regard, serosal involvement and dissemination of this tumor appears to be profoundly governed by a vicious triad of inflammation, vascular hyperpermeability, and new vessel formation, mechanisms that may be of lesser importance in solid tumor progression, where stimulus-independent tumor growth and evasion of apoptosis may be more pivotal [29]. In this regard, the ability of tumor cells to induce a MPE differs between various tumor types [15] and may constitute an additional hallmark of adenocarcinoma. We show that this MPE-promoting phenotype of lung adenocarcinoma, heavily dependent on NF-*κ*B-controlled gene expression and not on NF-*κ*B-independent tumor growth [12-15], can be selectively targeted by tailored proteasome inhibition. The present and other lines of evidence suggest that targeting host-tumor interactions rather than tumor cell cycling may present an effective future therapeutic strategy against cancer [30]. Compared with their activity against solid tumors, these approaches may be more effective against MPE, which is largely governed by the paracrine effects of tumor on the host immune system and vasculature [2,9-15,26]. Indeed, in our hands tailored proteasome inhibition specifically targeted lung adenocarcinoma-induced MPE formation but not the growth of this tumor in solid form. The specific anti-MPE effects of bortezomib were also evident by the fact that it was only effective when MPEs were present and preventive administration provided no additional benefit over therapeutic delivery. Although bortezomib enhanced apoptosis of pleural and subcutaneous tumors, this pro-apoptotic effect was of minor magnitude. In addition, bortezomib-induced apoptosis was observed in both subcutaneous and pleural tumors, and thus did not present a plausible explanation of the specific anti-MPE effects of the drug. However, bortezomib treatment limited paracrine phenomena specifically linked with MPE formation, such as angiogenesis, vascular hyperpermeability, and inflammation. Down-regulation of these mechanisms via suppression of NF-*κ*B-dependent genes in tumor cells may provide a more accurate mechanistic insight into the anti-MPE functions of proteasome inhibition.

Bortezomib has been tested against human NSCLC and has been found to be ineffective [24,25]. However, our results suggest that the drug may be worth testing specifically against NSCLC-induced MPE. In this regard, trials against NSCLC included a majority of patients without MPE and a minority of patients with MPE [24,25]. In addition, end-points related to MPE control were not included in the design of these studies. Hence a possible favorable effect of bortezomib treatment against MPE may have gone undetected and, despite the negative results of the aforementioned significant trials, bortezomib may be still worth testing against human MPE.

During the last few decades, knowledge of biologic mechanisms of disease has expanded tremendously. However, therapeutic targeting of single disease culprits is limited by biologic diversity and redundancy, as well as cost [31]. In this regard, simultaneous suppression of multiple disease promoting pathways by small molecules would be advantageous [26]. In our hands, a proteasome inhibition regimen designed to target NF-κB activation of lung adenocarcinoma limited MPE formation by suppressing the expression of multiple paracrine mediators of intrapleural adenocarcinoma dissemination and fluid extravasation, including TNF and CCL2 [14,15]. Bortezomib treatment blocked the constitutive and inducible NF-*κ*B activation of mouse lung adenocarcinoma, leading to suppressed MPE-related inflammation, angiogenesis, and vascular leakiness, and hence providing for a microenvironment less permissive for MPE development [9-15].

## Conclusions

Herein we showed the specific effects of NF-κB-tailored proteasome inhibition against MPE formation by mouse lung adenocarcinoma. In addition to previous genetic interventions, the pharmacologic approach employed herein strengthens the available data that support that serosal effusion formation, a hallmark of adenocarcinoma, is governed by expression of paracrine inflammatory and vasoactive mediators. Finally, we showed that tailored bortezomib treatment is highly effective against experimental MPE, the mouse analogue of a very common, difficult to treat, and debilitating occurrence in patients with cancer, setting a rational framework that supports the feasibility of clinical triage of proteasome inhibition against MPE.

## Competing interests

The authors declare that they have no competing interests.

## Authors' contributions

GTS conceived the main idea of the study. IP, TSB, IK, and GTS designed experiments. IP, SPK, CM, TPS, AK, SM, PT, and GTS carried out experiments. IP and GTS analyzed the data. IP and GTS wrote the paper. TSB and GTS contributed analytical tools and reagents. CR, TSB, IK, and GTS edited the paper. All authors reviewed and approved the final version of the manuscript.
